# Improved use of faecal immunochemical tests for haemoglobin in the Scottish bowel screening programme

**DOI:** 10.1177/09691413231175611

**Published:** 2023-05-25

**Authors:** Jayne Digby, Callum G Fraser, Gavin Clark, Craig Mowat, Judith A Strachan, Robert JC Steele

**Affiliations:** 1 573143 Centre for Research into Cancer Prevention and Screening, University of Dundee, Dundee, Dundee, Scotland, UK; 2 9571 Public Health Scotland, Edinburgh, Scotland, UK; 3 Department of Gastroenterology, Ninewells Hospital, Dundee, Scotland, UK; 4 Blood Sciences and Scottish Bowel Screening Laboratory, 59805 Ninewells Hospital and Medical School, Dundee, Scotland, UK

**Keywords:** Colorectal cancer screening, faecal immunochemical test, faecal haemoglobin, sex differences

## Abstract

**Objectives:**

This study aimed to develop a risk-scoring model in the Scottish Bowel Screening Programme incorporating faecal haemoglobin concentration with other risk factors for colorectal cancer.

**Methods:**

Data were collected for all individuals invited to participate in the Scottish Bowel Screening Programme between November 2017 and March 2018 including faecal haemoglobin concentration, age, sex, National Health Service Board, socioeconomic status, and screening history. Linkage with The Scottish Cancer Registry identified all screening participants diagnosed with colorectal cancer. Logistic regression was performed to identify which factors demonstrated significant association with colorectal cancer and could be used in the development of a risk-scoring model.

**Results:**

Of 232,076 screening participants, 427 had colorectal cancer: 286 diagnosed following a screening colonoscopy and 141 arising after a negative screening test result giving an interval cancer proportion of 33.0%. Only faecal haemoglobin concentration and age showed a statistically significant association with colorectal cancer. Interval cancer proportion increased with age and was higher in women (38.1%) than men (27.5%). If positivity in women were mirrored in men at each age quintile interval cancer proportion would still have remained higher in women (33.2%). Moreover, an additional 1201 colonoscopies would be required to detect 11 colorectal cancers.

**Conclusions:**

Development of a risk scoring model using early data from the Scottish Bowel Screening Programme was not feasible due to most variables showing insignificant association with colorectal cancer. Tailoring the faecal haemoglobin concentration threshold according to age could help to diminish some of the disparity in interval cancer proportion between women and men. Strategies to achieve sex equality using faecal haemoglobin concentration thresholds depend considerably on which variable is selected for equivalency and this requires further exploration.

## Background

Screening for colorectal cancer (CRC) using tests for the presence of haemoglobin in faeces as the initial test, followed by colonoscopy for a positive test result, has been proven to reduce disease-specific mortality in population-based randomised controlled trials^1^ and many countries world-wide have instigated screening programmes based on this evidence. The initial studies of CRC screening were conducted using guaiac-based faecal occult blood tests (gFOBT), but these have been superseded by faecal immunochemical tests (FIT) for haemoglobin since, unlike gFOBT, these are specific for human haemoglobin, detect only colorectal bleeding, testing can be automated eliminating human error, and they can provide a quantitative estimate of faecal haemoglobin concentration (f-Hb). In addition, in a population screening context, they are associated with higher uptake than gFOBT owing to a simplified testing algorithm involving only one sample, a more user-friendly hygienic collection device and a perception that they are modern, scientific and more reliable.^2^ As a result, and after a large pilot evaluation,^3^ quantitative FIT was introduced into the Scottish Bowel Screening Programme (SBoSP) as the initial test for all aged 50–74 in November 2017,^4^ with the other countries of the UK following suit.^5^

FITs are available in qualitative and quantitative formats^6^ and, despite the quantitative ability of automated FIT systems, these are invariably used at a single threshold in programmatic screening. Often the f-Hb threshold is set to give a positivity determined mainly by colonoscopy capacity rather than clinical effectiveness; in Scotland, this has been currently set at ≥80 µg Hb/g faeces, although kept under careful review, with other countries of the UK also introducing high f-Hb thresholds in order to ensure adequate colonoscopy capacity to deal with participants with positive test results.

Currently, about half of all CRC cases in the screened population in Scotland are diagnosed during the 2-year interval between invitations, indicating that, at the current threshold, the test is only about 50% sensitive for CRC. Two very recent papers from the Netherlands clearly demonstrate how for those with a negative screening test result, f-Hb results closest to the threshold for positivity showed a strong association with future diagnoses of CRC both as interval cancers^7^ (IC: CRC diagnosed in the interval between screening invites following a negative screening test result) or in subsequent screening rounds,^8^ compared with very low or undetectable f-Hb. Clinical sensitivity for CRC in screening programmes using FIT can be improved by lowering the f-Hb threshold used to determine a positive test result but at the expense of clinical specificity. In other words, more diseases will be detected but with a disproportionate number of false positive test results leading to a greater burden on colonoscopy resources as well as the possible individual harms associated with unnecessary colonoscopy. Furthermore, using FIT at a single threshold for all participants does not take account of important factors that affect f-Hb, including, as we have shown, age and sex^9^ and deprivation as inferred from area of residence.^10^ Adjustments to the screening algorithm for these factors on an individual basis might have potential to enhance the predictive value of f-Hb for colorectal neoplasia. Data on IC are also available, which allows accurate estimation of the effect of different thresholds across a range of f-Hb.

Some examples of more intelligent approaches to the use of FIT in CRC screening have been explored, although often these have included variables that are not readily available in screening programmes such as those collected via questionnaires, for example about lifestyle (including diet, alcohol intake and smoking habits), as well as characteristics such as body mass index. A recent study from The Netherlands developed a logistic regression model using f-Hb, age and sex to calculate risk of advanced neoplasia.^11^ Individualised thresholds based on 96.9% specificity, ranging from 36.9 µg Hb/g faeces for a 50-year-old female to 9.5 µg Hb/g faeces for a 75-year-old male, were identified to give all participants a comparable risk of advanced neoplasia (CRC plus advanced adenoma: AA) prior to colonoscopy. However, the cohort used was screening naïve and the results may not be transferable geographically to a country like Scotland where a higher threshold of ≥80 µg Hb/g faeces is currently used, as are different FIT analytical systems, an important consideration since these do not give comparable numerical f-Hb data^12^ as we have also shown in the SBoSP.^13^ Another recently published study from Denmark showed that a risk scoring model incorporating f-Hb, age, and sex could provide a slight improvement in CRC and adenoma detection compared with FIT alone at a cut-off of 20 µg Hb/g faeces.^14^ Similar results had previously been shown using data from the National Health Service (NHS) Bowel Cancer Screening Programme in England, in which screening history was also incorporated into the model.^15^

Further to the interest in risk scoring models as a strategy to offer a tailored approach to bowel screening, an increased focus is also emerging around tailored f-Hb thresholds to generate sex equality in screening performance indicators, including positivity, positive predictive values (PPV), sensitivity and specificity. The recent review by Clark et al.^16^ summarises work undertaken to date on these strategies, showing that the impact depends greatly on the variable selected for equivalency between women and men.

The aim of this study was to use the data accumulated since the introduction of FIT in the SBoSP in order to assess the development of a more rational approach to estimating an individual's risk of harbouring CRC based on f-Hb, age, sex, deprivation as assessed by the Scottish Index of Multiple Deprivation (SIMD), NHS Board of residence and screening history. In this way, we anticipated that an individually targeted approach might be introduced into CRC screening programmes across the UK that would enhance the diagnostic yield of screening, without impacting negatively on colonoscopy services. A further objective was to investigate an appropriate approach to stratified f-Hb thresholds in order to reduce some of the existing sex inequality in the SBoSP.

## Methods

Data were collected for all individuals invited to participate in the SBoSP from the nationwide roll-out of quantitative FIT in November 2017 up to March 2018. Age, sex and NHS Board of residence are routinely available in the screening population; in Scotland, this can be achieved by using the ubiquitously applied Community Health Index number matched to each screening test result. Estimation of socio-economic deprivation using the SIMD 2016 was available for 99.9% of participants via postcode data. The screening history of each participant was also assessed so that each screening episode could be classified as prevalence or incidence. Linkage with the Scottish Cancer Registry data up to April 2021 was then performed to identify all CRC cases diagnosed either as a result of screening, or as IC arising within 2 years of a screening FIT test result with f-Hb < 80 µg Hb/g faeces.

All data analysis was performed using SPSS software (Version 27, SPSS Inc., Chicago, IL, USA). With a view to creating a risk scoring model for CRC, SPSS was used to randomly select approximately 50% of the sample to be allocated to the derivation cohort, with the remainder used as the validation cohort. A number of potential predictors of risk of CRC, together with interactions between variables, were initially assessed in the derivation cohort using univariable logistic regression. Age was assessed as a continuous variable in the first instance and also converted to a categorical variable in quintiles to assess which of the two formats might give a more effective risk score. Based on PPV for CRC, f-Hb categories of <10, 10–19.9, 20–39.9, 40–79.9 and ≥80 µg Hb/g faeces were investigated.

## Results

Between November 2017 and March 2018, 359,655 individuals were invited to participate in the SBoSP, 50.5% women and 49.5% men. Overall uptake, defined as return of FIT with a valid test result, was 64.5% giving a final study population of 232,076. The characteristics of this cohort are described in Table 1 with odds ratios (ORs) for CRC for each of the variables investigated. In the cohort taking part in screening, 427 cases of CRC were diagnosed, with 286 being screen-detected and 141 IC giving an interval cancer proportion (ICP) of 33.0%.

**Table 1.** Characteristics of the study population with ORs of variables for CRC.

	*n*	%	Total CRC	%	ORs for CRC (95% CI)
No. of invitees	359,655				
No. of participants	232,076	64.5%	427	0.2%	-
Sex					
Women	120,935	52.1%	223	0.2%	-
Men	111,141	47.9%	204	0.2%	1.00 (0.82–1.20)
Age (years)					
50–54	57,767	24.9%	58	0.1%	-
55–59	44,777	20.2%	57	0.1%	1.27 (0.88–1.83)
60–64	53,150	22.9%	109	0.2%	**2.05** (**1.49–2.81)**
65–69	36,886	15.9%	107	0.3%	**2.90** (**2.10–3.99)**
70–74	39,496	17.0%	96	0.2%	**2.42** (**1.75–3.36)**
SIMD quintile					
5 (least deprived)	53,710	23.2%	90	0.2%	-
4	53,473	23.1%	96	0.2%	1.07 (0.80–1.43)
3	48,169	20.8%	88	0.2%	1.09 (0.81–1.46)
2	41,701	18.0%	87	0.2%	1.25 (0.93–1.67)
1 (most deprived)	34,842	15.0%	66	0.2%	1.13 (0.82–1.55)
FIT result (µg Hb/g faeces)					
<10	202,233	87.1%	68	0.0%	-
10–19	10,076	4.3%	21	0.2%	**6.21** (**3.81–10.13)**
20–39	7418	3.2%	29	0.4%	**11.67** (**7.55–18.03)**
40–79	4814	2.1%	23	0.5%	**14.27** (**8.89–22.92)**
≥80	7534	3.2%	286	3.8%	**117.30** (**89.95–152.96)**
Participation in previous screening rounds					
Never participated in previous rounds	172,655	74.4%	43	0.0%	1.24 (0.90–1.71)
Past participant but not in previous round	16,875	7.3%	42	0.2%	**1.39** (**1.01–1.92)**
Participated in previous round	23,239	10.0%	310	1.3%	-
Not previously invited	19,307	8.3%	32	0.2%	0.77 (0.53–1.10)
Participated in previous round					
Yes	23,239	10.0%	310	1.3%	-
No	208,837	90.0%	117	0.1%	0.94 (0.83–1.06)
NHS Board					
Ayrshire and Arran	17,589	7.6%	27	0.2%	1.02 (0.59–1.75)
Borders	6,514	2.8%	13	0.2%	1.32 (0.68–2.58)
Dumfries and Galloway	7,552	3.3%	17	0.2%	1.49 (0.80–2.76)
Fife	16,526	7.1%	41	0.2%	1.64 (1.00–2.70)
Forth Valley	13,547	5.8%	28	0.2%	1.37 (0.80–2.35)
Grampian	25,495	11.0%	52	0.2%	1.35 (0.84–2.18)
Greater Glasgow and Clyde	45,999	19.8%	82	0.2%	1.35 (0.84–2.18)
Highland	16,534	7.1%	25	0.2%	-
Lanarkshire	27,514	11.9%	44	0.2%	1.06 (0.65–1.73)
Lothian	32,339	13.9%	59	0.2%	1.21 (0.76–1.93)
Orkney	1123	0.5%	3	0.3%	1.77 (0.53–5.87)
Shetland	1138	0.5%	3	0.3%	1.75 (0.53–5.79)
Tayside	18,939	8.2%	32	0.2%	1.12 (0.66–1.89)
Western Isles	1267	0.5%	1	0.1%	0.52 (0.07–3.85)

Note: Statistically significant ORs are shown in bold. CI: confidence interval; OR: odds ratio; CRC: colorectal cancer; SIMD: Scottish Index of Multiple Deprivation; NHS: National Health Service.

Univariate logistic regression analysis identified detectable f-Hb, i.e., ≥10 µg Hb/g faeces, increasing age, and non-participation in the previous screening round as having statistically significant ORs for an increased likelihood of a diagnosis of CRC (OR 1.39, 95% confidence interval (CI): 1.01–1.92). When participation in the previous screening round was compared with any non-participation in the previous round (invited but never participated in any previous rounds, first-time invitee or participated in the past but not in the previous round), this association ceased to exist (OR 0.94, 95% CI: 0.83–1.06). No significant relationship was found between CRC and sex or between CRC and NHS Board of residence.

**Figure 1.**
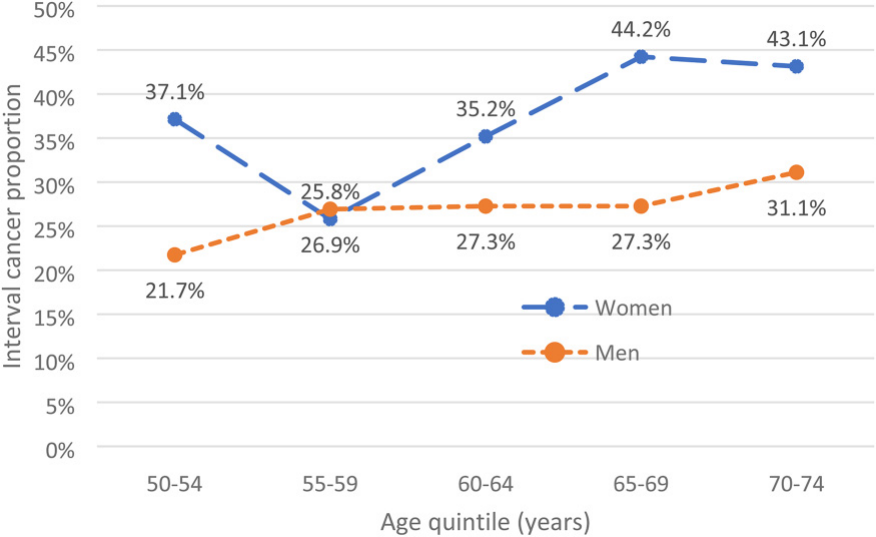
Interval cancer proportion (%) by age quintile (years).

Following randomisation of the population, the derivation cohort consisted of 115,739 individuals including 215 cases of CRC, 143 screen-detected and 72 arising as IC. The validation cohort consisted of 116,337 individuals including 212 cases of CRC, 143 screen-detected and 69 arising as IC. Univariate logistic regression analysis using the derivation cohort showed that only detectable f-Hb and increasing age retained a significant association with CRC. For this reason, and the striking ORs associated with high f-Hb, the development of a risk-scoring model was not feasible. Instead, further analysis of the full study population, derivation plus validation cohorts, was performed to investigate the effect of tailoring the f-Hb threshold in screening according to other risk factors. Table 2 describes the positivity and proportion of CRC detected by screening by age and sex. The proportion of CRC diagnosed in the 2-year interval following a negative screening test result, i.e., IC, increased with age and was higher in women than in men. Figure 1 shows a comparison of the ICPs between women and men for each age quintile.

**Table 2.** Positivity and proportion of colorectal cancer (CRC) detected by screening or arising as interval cancers by age quintile in women and men.

	*n*	*n* ≥ 80 µg Hb/g faeces	Positivity	Total CRC	Screen-detected CRC	Interval CRC	Interval cancer proportion
Women, age quintile (years)							
50–54	29,976	656	2.2%	35	22	13	37.1%
55–59	23,335	596	2.6%	31	23	8	25.8%
60–64	27,774	755	2.7%	54	35	19	35.2%
65–69	19,069	584	3.1%	52	29	23	44.2%
70–74	20,781	768	3.7%	51	29	22	43.1%
All	120,935	3359	2.8%	223	138	85	38.1%
Men, age quintile (years)							
50–54	27,791	758	2.7%	23	18	5	21.7%
55–59	21,442	726	3.4%	26	19	7	26.9%
60–64	25,376	954	3.8%	55	40	15	27.3%
65–69	17,817	815	4.6%	55	40	15	27.3%
70–74	18,715	923	4.9%	45	31	14	31.1%
All	111,141	4176	3.8%	204	148	56	27.5%

Table 3 shows the percentile ranks for each age quintile which would give the same positivity for women as for men when using the f-Hb threshold of ≥80 µg Hb/g faeces. Even when generating the same positivity as for men, women would still have had higher ICP. Using this strategy, an additional 1201 colonoscopies (an increase of 35.7% in women) would have been required to detect an additional 11 CRC cases via screening, with the assumption that the CRC was present at the time of the screening test.

**Table 3.** Effect of adjusting f-Hb threshold in women to equate to the positivity of men by age quintile on numbers of CRC above the threshold, IC proportions (%), number of additional colonoscopies required compared to a threshold of ≥80 µg Hb/g faeces, and number of additional ICs detected.

Age quintile (years)	f-Hb percentile to give women the same positivity as in men	f-Hb cut-off (µg Hb/g faeces)	CRC above threshold (screen-detected)	CRC below threshold (IC)	IC proportion	Additional colonoscopies compared with threshold ≥80 µg Hb/g faeces	Additional ICs detected compared with threshold ≥ 80µg Hb/g faeces
50–54	97.3	60.6	25	10	28.6%	154	3
55–59	96.6	54.0	23	8	25.8%	199	0
60–64	96.2	50.7	38	16	29.6%	300	3
65–69	95.4	45.0	31	21	40.4%	296	2
70–74	95.1	53.5	32	19	37.3%	252	3
All			149	74	33.2%	1201	11

f-Hb: faecal haemoglobin concentration; CRC: colorectal cancer; IC: interval cancer.

## Discussion

Development of a risk-scoring algorithm using early data from the SBoSP was not feasible due to a lack of variables showing significant association with CRC. Since increasing age was the only variable other than f-Hb with an association with CRC, we instead investigated the possibility of tailoring f-Hb thresholds in each age quintile to offset some of the inequality identified between women and men in terms of positivity and ICP. Our data show that even if identical positivity was generated in women and men according to age quintile, women would still have had a markedly higher ICP than men.

The reason for this sex inequality in cancer detection is not well understood. It has previously been demonstrated, from SBoSP data, that women have lower f-Hb than men.^17^ This is also true when comparing the median f-Hb of those diagnosed with CRC.^18^ As well as higher ICP, other indicators of poorer CRC outcomes are also more strongly associated with women including a higher percentage of emergency CRC presentations^19^ and later stage IC. A recent review of the existing literature around sex inequalities in CRC screening listed slower colonic transit time, longer colon length and more constipation in women as reasons why they might be disadvantaged in CRC screening compared with men, with greater opportunity for in vivo degradation of f-Hb.^16^ Moreover, studies on IC using gFOBT in CRC screening have also shown a disproportionate number of cases occurring in women with proximal lesions.^20,21^ A potential reason for this is explained in a review of the characteristics of IC^22^ which states that older age is associated with a higher prevalence of proximal lesions and, since women generally live longer than men, a higher number of elderly women will have proximal CRC. This has also been reflected in a very recent study from Belgium in a CRC screening programme using FIT,^23^ although the higher ICP in women was also shown to be independent of tumour location. Indeed, data from Scotland have shown that sex differences in median f-Hb persisted when compared at all CRC sites.^17^ ICs are also associated with the serrated pathway of carcinogenesis.^22,24^ Sessile serrated lesions occur more often in the proximal colon, and bleed less than adenocarcinoma and are therefore less likely to trigger a positive FOBT; these characteristics may contribute to some of the gender inequality in CRC detection using FOBT screening.

An increasing number of studies investigating the effect of stratified thresholds for women and men are emerging. In Finland, implementation of sex-based thresholds of 25 µg Hb/g faeces for women and 70 µg Hb/g faeces for men have led to similar CRC detection rates between the sexes.^25^ A Danish study investigating the impact of varying f-Hb cut-off by age and sex, where women aged 55–59 and 65–69 had a lower threshold than men of the same ages, found that overall sensitivity and specificity for neoplasia detection improved, but inequalities in these factors between the sexes would in fact increase and therefore other strategies should be considered.^26^ The review from Clark et al.^18^ presented preliminary data from the SBoSP showing that, to achieve equal positivity with men using ≥80 µg Hb/g faeces, an overall threshold for women of ≥50 µg Hb/g faeces would be required, and this would need to be lowered further to ≥40 µg Hb/g faeces in women to achieve equivalent ICP with men. Of several factors which could be selected to achieve equivalency between women and men in CRC screening, the authors of the review stated that positivity would be their favoured strategy owing to the fact that any effects could be rapidly assessed. In the present study, lowering f-Hb thresholds in women to give identical positivity as in men still resulted in a markedly higher ICP in women compared to men. For ICP to be similar between women and men, far lower f-Hb thresholds for women would need to be adopted. In countries in which colonoscopy capacity is limited, such as in Scotland, lowering the threshold used for women while maintaining the threshold of ≥80 µg Hb/g faeces for men, so that men would not be disadvantaged, would generate many more additional false-positive screening test results; the implications of this must be carefully considered in terms of limiting exposure of participants to unnecessary colonoscopy, and the financial cost and implications on resources. The strategy explored in the present study would have resulted in a 15.9% increase in the number of colonoscopies required, which translates as an additional 2226 colonoscopies annually in Scotland. The effect of using a threshold of 40 µg Hb/g faeces for women compared with 80 µg Hb/g faeces for men in Sweden was that women had a significantly lower PPV for CRC than men, with more false positive test results, but it was concluded that the increase in CRC detection outweighed the additional screening costs.^27^ A Finnish study including measures of cost-effectiveness also supported lower thresholds in women than men when restrictions on colonoscopy capacity and costs are in place,^28^ but these results may not be transferable to other countries; similar analyses, perhaps in a randomised controlled trial, would be required before full implementation of a similar approach in the SBoSP.

An interesting observation is that using lower f-Hb thresholds to give equivalent positivity for women as for men would have had the largest effect on the proportion of cancers detected by screening in the youngest age quintile, where the ICP would have potentially fallen from 37.1% to 28.6%, despite this group having the lowest decrease in stratified f-Hb threshold. Although the number of CRC cases is relatively small, the disadvantages women experience in CRC screening programmes may be greatest at the younger end of the screening age range, whereas for older age groups, lowering the threshold may result in a larger number of false positives. This theory requires further exploration on a larger scale.

A strength of this study was that a large cohort was available with full linkage for IC. One limitation of this study is that data were not available on AAs as potential precursors to CRC. With one aim of colorectal screening being early detection of CRC and precursor lesions, inclusion of these data would have allowed a more thorough assessment of the feasibility of developing a neoplasia risk scoring model for use in the SBoSP. Our conclusions around the significant burden of false positive test results if lowering the f-Hb threshold for women do not consider that a greater number of AAs would likely be detected in women using this strategy, also potentially having a positive follow-on effect on ICP. In addition, calculations for the impact on ICP, when adjusting the f-Hb threshold, are estimates due to the assumptions being made about the presence of malignancy at the time of the screening round.

In addition to strategies to reduce the ICP such as the f-Hb threshold adjustment investigated in this study, it is important to also address other types of missed CRC in the screened population. Around 25% of those with a positive screening test result in the SBoSP do not attend for follow-up colonoscopy. Since these participants would be expected to have a greater risk of CRC than the additional women who would be invited for colonoscopy if sex-stratified f-Hb thresholds were to be adopted, it may be that a focus on improved uptake of colonoscopy could be a more cost-effective strategy to increase detection of early-stage CRC. In addition, it might be that greater gains in CRC detection could be achieved through an increased focus on improving the performance of colonoscopists^29^ to ensure sufficient visualisation of the colon is achieved, along with full resection of lesions to reduce the number of post-colonoscopy CRC cases.

## Conclusions

Tailoring the f-Hb threshold in CRC screening programmes is an option for reducing some of the inequality experienced by women. However, an interesting conundrum for screening programme organisers in this context is which of the several possible variables to select for equivalency, such as positivity, ICP, PPV, sensitivity, specificity, CRC risk, etc. Published data from The Netherlands,^9^ Denmark,^23^ Sweden^25^ and Finland^26^ along with unpublished data from Scotland^16^ and the results of this current study show exploration of some of these options. Considerations must also be made around how to evaluate the impact of such a strategy depending on which variable is chosen. Generating equivalent positivity in women and men, as evaluated in this study according to age quintile, is one strategy for which the impact could be relatively easily and rapidly assessed. Selecting f-Hb thresholds to achieve similar ICPs in women and men is an option that would require far lower f-Hb thresholds than would equivalent positivity. Moreover, the nature of ICs means that this option would need a much longer evaluation period, possibly over several screening rounds. Tailoring the f-Hb threshold to achieve equal specificity is a further option, but one which may not have as significant an impact at higher f-Hb thresholds.^11^ Therefore, opportunities for tailoring the f-Hb threshold to reduce inequalities are plentiful but further work in validating these approaches on a larger scale along with modelling around cost-effectiveness would be required prior to implementation. Furthermore, although f-Hb threshold adjustment could increase the number of screen-detected cancers, a large number of unnecessary colonoscopies would be required with associated costs and risks to participants. Therefore, a better option may be to focus efforts on colonoscopy uptake and quality. Further comparison studies are required.
